# Disentangling the Role of the MEC and LEC in the Processing of Spatial and Non-Spatial Information: Contribution of Lesion Studies

**DOI:** 10.3389/fnsys.2017.00081

**Published:** 2017-10-27

**Authors:** Etienne Save, Francesca Sargolini

**Affiliations:** ^1^Laboratory of Cognitive Neuroscience, Aix Marseille University, CNRS, LNC UMR 7291, Marseille, France; ^2^Institut Universitaire de France, Paris, France

**Keywords:** entorhinal cortex, spatial cognition, lesions, rats

## Abstract

It is now widely accepted that the entorhinal cortex (EC) plays a pivotal role in the processing of spatial information and episodic memory. The EC is segregated into two sub-regions, the medial EC (MEC) and the lateral EC (LEC) but a comprehensive understanding of their roles across multiple behavioral contexts remains unclear. Considering that it is still useful to investigate the impact of lesions of EC on behavior, we review the contribution of lesion approach to our knowledge of EC functions. We show that the MEC and LEC play different roles in the processing of spatial and non-spatial information. The MEC is necessary to the use of distal but not proximal landmarks during navigation and is crucial for path integration, in particular integration of linear movements. Consistent with predominant hypothesis, the LEC is important for combining the spatial and non-spatial aspects of the environment. However, object exploration studies suggest that the functional segregation between the MEC and the LEC is not as clearly delineated and is dependent on environmental and behavioral factors. Manipulation of environmental complexity and therefore of cognitive demand shows that the MEC and the LEC are not strictly necessary to the processing of spatial and non-spatial information. In addition we suggest that the involvement of these sub-regions can depend on the kind of behavior, i.e., navigation or exploration, exhibited by the animals. Thus, the MEC and the LEC work in a flexible manner to integrate the “what” and “where” information in episodic memory upstream the hippocampus.

## Introduction

The segregation of the entorhinal cortex (EC) into two main sub-regions, the medial entorhinal cortex (MEC) and the lateral entorhinal cortex (LEC), is classically established in the rat (Krieg, 1946; Blackstad, 1956; Burwell and Amaral, 1998b; Sewards and Sewards, 2003; Kerr et al., 2007). In the frame of the influential theory of two distinct processing pathways in the medial temporal lobe, it has been proposed that the MEC is part of the dorsal pathway (processing “where”, i.e., spatial, information) whereas the LEC is part of the ventral pathway (processing “what”, i.e., non-spatial, information; Mishkin et al., 1983). Both pathways converge within the hippocampus that would combine spatial and non-spatial information to form representations that underlie episodic memory. As detailed in numerous experimental and review articles (see for example: Witter, 1993; Burwell and Amaral, 1998a,b; Dolorfo and Amaral, 1998a,b; Sewards and Sewards, 2003; Kerr et al., 2007; van Strien et al., 2009; Agster et al., 2016; Tomás Pereira et al., 2016), the MEC and the LEC are characterized by a different pattern of connectivity which suggests a functional segregation. The MEC receives strong projections from the postrhinal cortex, whereas the LEC is mainly connected with the perirhinal cortex (Insausti et al., 1997; Burwell and Amaral, 1998a). Occipital, parietal and cingulate areas are more heavily connected to the MEC, whereas the insular and prelimbic and infralimbic frontal areas are more heavily connected to the LEC (Burwell and Amaral, 1998b). Although this issue will not be developed in this review, it is worth noting that there are extensive projections from olfactory regions, i.e., the olfactory bulbs and the piriform cortex to the LEC. Both MEC and LEC have strong reciprocal connections with subcortical structures, such as the thalamus, the amygdala, the claustrum and the septum (Alonso and Köhler, 1984; Wouterlood, 1991; Pitkänen et al., 2000; Kitanishi and Matsuo, 2017; Reviews by Furtak et al., 2007; van Strien et al., 2009). For each sub-region, there are also substantial disparities in the pattern of connectivity (i.e., proportion of connections from and to the hippocampus, cortical areas and subcortical nuclei) across the entorhinal-to-dentate gyrus projecting medial, intermediate and lateral bands (Kerr et al., 2007). These three bands spans the MEC and LEC and longitudinal intrinsic connections remain confined within each band (Dolorfo and Amaral, 1998b) allowing portions of the MEC and LEC in the same region to be interconnected (van Strien et al., 2009). Those interconnected portions of the LEC and MEC give rise to a topologically arranged circuitry between the EC and the hippocampus as the dorsolateral band is connected to the dorsal part of the hippocampal formation, the intermediate band to the intermediate part, and the medial band to the ventral part (Canto et al., 2008). Thus, the interaction between the MEC and the LEC via associational connections would allow an integration of spatial and non-spatial information at the entorhinal level.

It is relatively recently, in particular in comparison to the hippocampus, that the issue of the respective function of the MEC and LEC has been addressed. Noticeably, lesion studies accumulated over the past 15 years have largely contributed to the hypothesis of a MEC-mediated-where pathway that processes spatial information and a LEC-mediated-what pathway that processes non-spatial information. Electrophysiology studies have supported this hypothesis by showing that the MEC contains grid cells with multiple firing fields that are arranged in an hexagonal grid pattern (Fyhn et al., 2004; Hafting et al., 2005), as well as other spatially-modulated cells (i.e., head-direction cells; Sargolini et al., 2006; border cells: Savelli et al., 2008; Solstad et al., 2008). In contrast, LEC neurons show little spatial selectivity compared to the MEC (Hargreaves et al., 2005; Yoganarasimha et al., 2011; Tsao et al., 2013). Furthermore, the discovery of grid cells in the MEC (Hafting et al., 2005) but not in the LEC have suggested that the function of the two sub-regions can be dissociated with respect to the category of cues, allothetic (i.e., external or environmental) or idiothetic (i.e., internal or self-motion) they process.

More recently, the notion that the MEC and LEC are functionally separate entities has been questioned. It is very likely that there is cooperation and functional interaction between the two sub-regions. This is consistent with the existence of intrinsic connectivity, although its relationship to EC processing is still poorly known (Canto et al., 2008). The hypothesis of a functional interaction between the two sub-regions comes from lesion (e.g., Hunsaker et al., 2013; Van Cauter et al., 2013; Wilson et al., 2013; Chao et al., 2016), imaging (Beer et al., 2013), and electrophysiological (Deshmukh and Knierim, 2011; Tsao et al., 2013) studies. Another aspect that it is worth mentioning is that both the MEC and the LEC only partly contribute to hippocampal place cells activity. MEC lesions do not abolish place cell firing, but affects place field stability (Hales et al., 2014). Conversely, LEC lesions abolish hippocampal rate remapping following changes in the shape or the color of the environment suggesting an interaction between spatial and non-spatial processing (Lu et al., 2013). Together these studies suggest that the hippocampal and the MEC place coding systems are relatively independent, and that both the MEC and the LEC are able to influence the hippocampal place cell system.

Thus, based on these recent findings contradicting the dual stream hypothesis, a strict functional dichotomy between the MEC and the LEC is no longer sustainable. In particular the spatial vs. non-spatial distinction is not as clear-cut as expected from the theory. In this article we show that there are lesion studies providing contradictory results regarding the specific role of the MEC and the LEC in processing spatial and non-spatial information. We first review data showing an implication of the MEC but not the LEC in both idiothetic cue-based (path integration) and allothetic cue-based navigation. We then show that the implication of the MEC in allothetic navigation depends on the system of reference, local (based on proximal landmarks) or global (based on distal landmarks) used by the animals (Benhamou, 1997). Finally, we review data on the effects of MEC and LEC lesions in spontaneous object exploration tasks, showing a large overlap between MEC and LEC functions.

This led us to the conclusion that the MEC and the LEC have distinct functions but this functional dissociation can be modulated by the behavior (navigation vs. exploration) and/or cognitive demand. First, different behaviors such as goal-directed navigation and exploration may result in different involvement of the MEC and LEC for the processing of distal landmarks and proximal landmarks. Second, the two sub-regions may be involved when the cognitive demand is high (for example when the animals have to process a large amount of information or when they need to form complex associations between stimuli). In that case, the “what” and “where” information are integrated upstream the hippocampus in a coherent representation so as to correctly process information and adapt the behavioral response to a specific context.

## EC and Navigation: Idiothetic vs. Allothetic

The MEC/LEC functional dichotomy is largely supported by studies investigating the impact of EC lesions in navigation abilities using idiothetic information (i.e., motion-related information essentially provided by vestibular, proprioceptive, somatosensory information and visual flow) or allothetic information (i.e., environmental information provided by all sensory systems). Rats are consistently impaired in both idiothetic and allothetic navigation following MEC lesions, and consistently non impaired following LEC lesions.

### Idiothetic Navigation

Both allothetic and idiothetic cues are generally available to the animal but it can happen that allothetic information are made irrelevant or unavailable, for example in darkness. The animal can nevertheless use idiothetic cues to maintain minimal navigational ability. This strategy called path integration allows the animal to track its position relative to a reference place and consists in continuously integrating motion-related information during linear and angular acceleration to generate a vector indicating the distance and direction to this place. Path integration can be tested in a homing task in which the animal has to return to its “nest” after a circuitous outward path leading to a (not visible) food location. In this task, allothetic information are made irrelevant or eliminated so to leave available only idiothetic cues.

More strikingly than for any other cortical region, there is strong evidence that the MEC plays a role in path integration. Idiothetic information is conveyed to the MEC via multiple parallel pathways involving subcortical structures and cortical areas (Rochefort et al., 2013; Hitier et al., 2014; Jacob et al., 2014). We have shown that lesions covering the entire EC or restricted to the MEC subdivision impaired rat performance in a homing task (Parron and Save, 2004; Van Cauter et al., 2013). In contrast, LEC lesions had no effect in this task (Van Cauter et al., 2013).

To further investigate the role of the MEC in path integration, we examined whether the MEC is necessary for the integration of linear movements, one of the two components of path integration (the other being the integration of angular movements). Rats were trained in a novel distance estimation task in which they had to reproduce several distances on a linear track in the absence of any external landmarks. MEC excitotoxic lesions affected the ability of rats to perform such estimation, suggesting that the MEC is necessary for linear integration (Jacob et al., 2017).

The discovery of grid cells in the dorsal MEC revealed the potential neural machinery underlying path integration. The regular organization of grid cells firing fields has been suggested to arise and be maintained by the use of idiothetic cues (McNaughton et al., 2006). There is nevertheless few evidence that grid cells are involved in this strategy. Allen et al. (2014) have shown that mice lacking Glu-A1 subunit of the AMPA receptors had altered grid cell field organization and were impaired in a path integration task. We have recently addressed this question indirectly, by investigating the effects of medial septum (MS) inactivations in the self-motion-based distance estimation task (see above). Indeed, silencing MS activity has been shown to reduce theta oscillations in the MEC and to disrupt the grid-like firing pattern of the grid cells (Brandon et al., 2011; Koenig et al., 2011), consistent with theoretical and experimental studies showing a tight connections between grid cells activity and theta oscillations (Burgess et al., 2007; Hasselmo et al., 2007). We found that MS inactivations provoked deficits in distance estimation that were similar to those induced by MEC lesions (Jacob et al., 2017). These results confirm previous findings on the involvement of the MS in path integration (Martin et al., 2007) and point to MEC theta oscillations and grid cells as the neural substrate of path integration.

### Allothetic Navigation

The ability to navigate using allothetic cues is generally addressed using the Morris Water Maze task, in which rats learn to find a submerged platform in a circular pool using distal landmarks (Morris et al., 1982). A large amount of studies from the 80s to the 90s have investigated the effects induced by complete EC lesions (covering both the MEC and the LEC) in this task, and have shown contradictory results. As emphasized by Morrissey and Takehara-Nishiuchi (2014) in a recent review, most of this discrepancies may be accounted for by a variability in lesion extent. In all cases, deficits are observed when the most caudal and medial part are damaged. In the late 90 s, a study from Ferbinteanu et al. (1999) showed that electrolytic lesions of the medial perforant path yielded a place learning deficit, whereas lesion of the lateral perforant path preserved performance, thus suggesting a dissociation between the MEC and the LEC (Ferbinteanu et al., 1999).

Recent studies specifically tested the contribution of the MEC and the LEC to place navigation and found that lesions of the MEC consistently impaired performance in the Morris water maze task. The impairment was particularly evident when lesions included the most dorsal and caudal parts of the MEC (Steffenach et al., 2005; Van Cauter et al., 2013; Hales et al., 2014). In line with these results, we also showed that selective MEC lesions delayed the acquisition of the platform location and impaired retention in the probe test. In contrast, LEC-lesioned animals did not differ from control rats, thus supporting the functional dichotomy between the two areas (Van Cauter et al., 2013). It is important to note that MEC-lesioned animals tested in the water maze were the same that showed impairment in path integration. This suggests that the MEC has a major role in spatial navigation based on both an allothetic and an idiothetic reference frame. A main question is whether these two processes rely on the same neural substrate within the MEC. Given the putative role of the grid cells in path integration, it is important to know whether these neurons are also involved in place navigation. The fact that their grid-like firing pattern is influenced by external landmarks (Barry et al., 2007; Krupic et al., 2015) suggests that MEC grid cells are not exclusively involved in path integration but may also participate to place navigation. Their specific contribution to this process remains to be determined.

## EC and Navigation: Proximal vs. Distal

**Figure 1:**
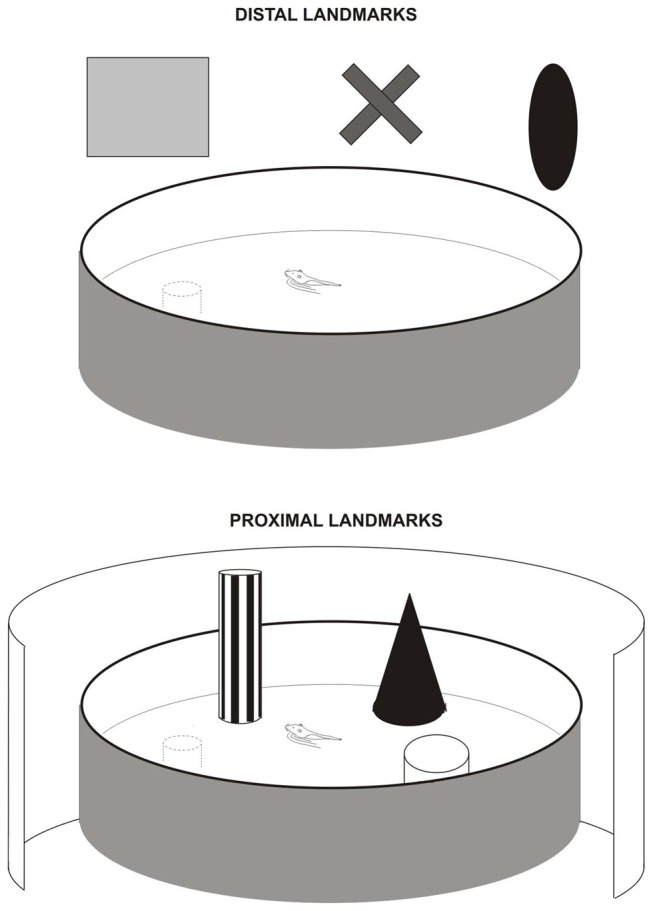
Navigation task in the water maze with distal landmarks (upper figure) or proximal landmarks (lower figure) used by Save and Poucet (2000) and Parron et al. (2004). In the distal cue condition, all room cues were available. In the proximal condition, the pool was surrounded by an opaque curtain to mask room cues. The proximal landmarks were three distinct objects placed in the water near the wall.

Allothetic navigation is based on the use of external landmarks that can be proximal or distal. In laboratory experiments, we generally refer to distal cues as bi-dimension visual cues located out of the animal’s locomotor space (extra-maze cues), and proximal cues as tri-dimension objects with multi-sensory characteristics, located in the animal’s locomotor space and that can be directly approached and explored (intra-maze cues). In the Parron et al. (2004) study we tested the effects induced by complete EC lesions (including MEC and LEC) in two versions of the Morris Water Maze task (Figure 1). One version was the* distal cue condition*, the “classical” version, in which rats had to use remote room cues to locate the goal. The second version was the *proximal cue condition* in which rats were required to use three objects directly placed in the pool to locate the platform. Rats with EC lesions were not able to learn the task using distal landmarks but were able to do it using proximal landmarks. Consistent results were provided in a recent study (Hébert et al., 2017), investigating the effects induced by genetic of pharmacological blockade within the EC of an extracellular protease (the tissue plasminogen activator—tPA), in two spatial tasks: the Morris Water maze task and a 2-trial place recognition task in a T-maze. Intra-EC inactivation of the tPA provoked deficits in both tasks when the animals had to use distal landmarks. In particular, animals showed delayed acquisition of the place navigation task in the distal condition. No deficit was found however in the proximal condition suggesting that the tPA in neither the MEC nor the LEC is necessary for the use of proximal landmarks. Using a place navigation task similar to Parron et al. (2004), Van Cauter et al. (2013) found a MEC-LEC dissociation: MEC lesions induced a mild deficit whereas LEC lesions did not affect the use of distal landmarks. Together these results point to a role of the EC in the processing of distal landmarks, probably through the activation of the MEC and not the LEC. In contrast, the processing of proximal landmarks does not seem to require both the MEC and the LEC. Further data are needed to support this hypothesis however.

These findings are not fully consistent with the hypothesis that the MEC and the LEC are involved in establishing a global and a local spatial reference frame, respectively. Both frameworks are indeed necessary to correctly navigate in any given environment. In particular, it has been proposed that distal cues are necessary to set the internal spatial coordinate system, whereas local cues are required to adapt such coordinate system to a particular environment (Knierim and Hamilton, 2011). The properties of the different space-coding cells in the MEC (and in particular the grid cells and the head-direction cells) are consistent with a specific involvement of the MEC in establishing a global reference frame (Hafting et al., 2005; Sargolini et al., 2006). Also in the case of the LEC, lesion and electrophysiological data strongly support an involvement of this area in establishing a local spatial framework (Knierim et al., 2013; Neunuebel et al., 2013; Kuruvilla and Ainge, 2017). The fact that LEC lesions (or complete EC lesions) do not impair animal performance in a place navigation task based on proximal landmarks (Parron et al., 2004) contradicts this hypothesis. However, one possibility is that navigation based on proximal cues does not involve the processing of complex associations between the context and the items that are contained in the context (such as the intra-maze objects), but may involve simple item recognition processes together with the integration of motion signals. Since the LEC does not seem to be required for simple object recognition (see next paragraph), this may explain why LEC lesions spared rat performance in the proximal cue condition of the water maze. In addition, this hypothesis is coherent with the observation that rats with associative parietal cortex lesions exhibited strong deficits selectively in the proximal cue condition of the water maze (Save and Poucet, 2000), and neurons in the parietal cortex are modulated by both landmarks and movement (Wilber et al., 2014). The circuit may also involve the retrosplenial cortex, which has been suggested to combine idiothetic cues and allothetic cues (Alexander and Nitz, 2015). Interestingly, processes in the APC may be independent on those occurring in the MEC (Whitlock, 2014), thus suggesting that navigation based on proximal and distal landmarks are two dissociated processes.

## EC and Object Exploration

Exploration is essential to acquire knowledge about the environment and adapt to changes, and hence results in “converting” novelty into familiarity. In intact rats, repeated exposure to an environment containing various objects first triggers intense exploration of these objects and eventually lead to habituation of locomotor activity and object exploration. When a change suddenly occurs in the environment, for example an object “appears” at a new location, the animal exhibits re-exploration targeting the source of the modification, i.e., the displaced object, relative to objects that have not been displaced (Save et al., 1992). Exploration dynamics therefore reflects learning of the environmental characteristics (spatial and non-spatial), which is supposed to result in the formation of episodic-like memory.

A number of studies have been done using this paradigm to investigate the ability of lesioned rats to integrate the spatial, i.e., object location and non-spatial, i.e., object recognition, components of episodic memory. In these studies, spatial change is produced by modifying a configuration of objects located in the environment. One object is displaced to a new location while one or several other objects remain at their initial location. Parron et al. (2004) have shown that rats with complete EC lesions (including the MEC and the LEC) are not able to detect the spatial change (one displaced object and three non displaced objects) and therefore do not re-explore the displaced object more than the non-displaced objects. Interestingly, when a non-spatial change was applied, i.e., a familiar object was replaced by a novel object, EC-lesioned rats were also impaired. To clarify the contribution of the MEC and the LEC to the deficits, rats with MEC or LEC lesions were submitted to a similar procedure (Van Cauter et al., 2013). MEC rats were impaired to detect the spatial change but were able to detect the non-spatial change. In contrast the LEC rats were unable to detect the spatial change and the non-spatial change. These results suggest that the MEC is exclusively required for processing spatial information, whereas the LEC is involved in both spatial and non-spatial information processing. Electrophysiological data are also consistent with this result. Neurons in the LEC display firing fields close to the current or the former locations of objects in the environment (Deshmukh and Knierim, 2011; Tsao et al., 2013). In addition, neurons encoding objects and position were found both in the MEC and the LEC (Keene et al., 2016). Similarly, early gene mapping studies showed that the LEC (and the MEC) is activated during spatial and non-spatial tasks (Beer et al., 2013). It is nevertheless intriguing that in a simple version of the object exploration task in which only two objects were present (i.e., one displaced object and only one non-displaced object), LEC rats exhibited control-like performance in spatial and non-spatial recognition (whereas MEC rats were still impaired in the processing of spatial information; Van Cauter et al., 2013). This result is in accordance with recent studies showing no impairment in simple object recognition tasks following LEC lesions. In contrast LEC-lesioned animals exhibited decreased performance when animals had to associate a specific object with a specific context (Wilson et al., 2013). This suggests that the role of the LEC in recognition memory goes beyond simple item recognition and that the LEC is critical for associative memory. In particular, the LEC appears to be involved in the representation of more complex object identity × context (Hunsaker et al., 2013; Wilson et al., 2013; Kuruvilla and Ainge, 2017) or even object identity × context × location (Chao et al., 2016). Overall, these results suggest that the LEC plays a role in the combination spatial and non-spatial aspects of the environment, which is essential to the formation of episodic memory.

**Figure 2:**
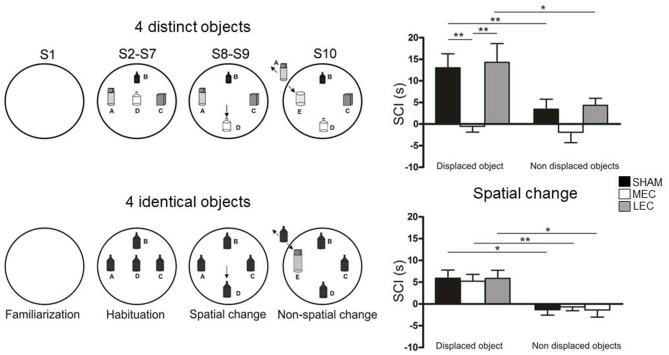
Object exploration task used by Rodo et al. (2017). (A) The rats were submitted to 10 successive 4-min exploration sessions (S1-S10) in a circular arena containing four distinct or four identical objects (A–D present from S2 to S10). In both conditions, during S8 and S9, one object (B as indicated by the arrow) was moved to a new location (spatial change). During S10, one familiar object (A) was replaced by a novel object (E as indicated by the arrow). (B) The ability of rats to detect spatial novelty was measured using a spatial change index (SCI = duration of object exploration in S8-S7) calculated for the displaced object and the non-displaced objects. Positive bars indicate a re-exploration of the objects. SHAM and lateral entorhinal cortex (LEC) rats re-explored the displaced objects but not the non-displaced objects indicating preserved ability to process spatial information. In contrast the medial entorhinal cortex (MEC) rats did not re-explore either kind of object indicating that they were not able to process spatial information. LEC rats were impaired to identify the novel object (S10) in the four distinct object condition but objet recognition ability was restored in the four identical object condition (data not shown). MEC and SHAM rats were able to detect the novel object in the two conditions. *p < 0.05, **p < 0.01.

Combining spatial and non-spatial information should be more or less demanding depending on the amount of objects available in the environment. We hypothesized that increasing the number of objects would result in a higher cognitive demand because the animals have to process a large amount of information, manage a high working memory load, and process complex associations between objects. This hypothesis is in line with the work of a number of authors who have previously proposed that environmental complexity increases with the number and/or diversity of objects placed in the environment (Berlyne, 1960; Hughes, 1997, 2007). We recently addressed the hypothesis that MEC and LEC functions may be modulated by environmental complexity according to Berlyne’s and Hughes’ conception. Accordingly, we used the behavioral procedure developed by Sannino et al. (2012) and Olivito et al. (2016) and we investigated the effect of reducing the number (from 4 to 3) and/or diversity of objects on MEC and LEC involvement in spatial and object recognition using the Van Cauter et al.’s (2013) object recognition task (Rodo et al., 2017; Figure 2). Rats with MEC lesions were not able to detect the spatial change when the four objects were different but this ability was restored when they were all identical. Similarly, rats with LEC lesions were impaired to detect the non-spatial change when the four objects were different but this ability was restored when all objects were identical or the number of different objects was decreased (from 4 to 3). Thus, these results indicate that the MEC and the LEC are not absolutely necessary for processing spatial and non-spatial information and that the involvement of the two regions can be modulated by environmental factors. Consistent with this finding, Kuruvilla and Ainge (2017) showed that the involvement of the LEC in object recognition depended on the number of objects located in the environment suggesting that the LEC is involved when the animal has to perform a complex task (four objects) but not a simpler task (two objects). These results are also coherent with a recent study from Ku et al. (2017) that showed a greater involvement of the LEC as a function of the number of items to be processed (5 vs.10 odors) in an odor recognition task.

These results also implicate that alternative pathways are recruited to restore the processing capacities in lesioned rats. One possibility is that each sub-region can compensate to some extent, i.e., as far as the amount of information is limited, for the defection of the other. This is possible since the MEC and the LEC share a number of cortical inputs. For example, in addition to their main target, the perirhinal cortex projects to the MEC and the postrhinal cortex projects to the LEC thus allowing both the MEC and the LEC to receive spatial and non-spatial information (van Strien et al., 2009).

Thus, the functional organization of the EC system appears to be more complex than the structure-function dissociation between MEC-spatial and LEC-non-spatial. The MEC is mainly involved in spatial processing but may be able in some conditions to process also non-spatial information. Similarly, the LEC is mainly involved in non-spatial processing but may be able to process spatial information. According to this hypothesis, we expect an important role of MEC/LEC functional interactions via local connections.

## Conclusion

The functional organization of the EC is still poorly known and a global theory is not yet at hand. Available data suggest that the EC underlies multiple functions that are mediated by different circuits. Basically, a distinction can be made between a neural system devoted to the processing of allothetic information and a system devoted to the processing of idiothetic information. The allothetic system would involve both the MEC and LEC whereas the idiothetic system would be channeled only through the MEC. Whether and how these systems interact remains to be investigated. Considering the allothetic system, a distinction based only on the MEC and the LEC is not sufficient to account for the behavioral effects observed after lesioning either one or the other structure. Some authors have suggested to distinguish between a MEC-system processing information within a distal frame of reference and a LEC-system processing information within a proximal frame of reference (Knierim and Hamilton, 2011) but again, this distinction does not match fully the MEC-LEC functional dissociation. We suggest that an important source of modulation for the contribution of the MEC and LEC is the behavior. Indeed the effects of MEC lesions in navigation based on proximal landmarks are not consistent with those observed in the object exploration task. Thus, goal-directed navigation and exploration may result in different involvement of the MEC and LEC for the processing of distal landmarks and proximal landmarks. Consistent with this idea, Yoo and Lee (2017) have recently shown that the involvement of MEC in visual scene processing (involving the processing of distal visuospatial information) is dependent on the kind of behavioral response used by the animals.

Considering the role of the EC in episodic memory, a main dichotomy between the MEC-spatial and LEC-non spatial is classically proposed (Knierim et al., 2013). We concur with the proposal that although the general hypothesis is not to be rejected, the notion of a strict dichotomy needs to be revised (Knierim et al., 2013). To complement this hypothesis we suggest that the involvement of the MEC and LEC in the processing of spatial and non-spatial information depends on the task demand (Rodo et al., 2017). In conditions of “simple” environments that do not require to process a large amount of information or complex associations between stimuli the MEC and LEC may not be necessary for spatial and non-spatial processing. The notion that cognitive demand is an important factor that modulates the involvement of the MEC and LEC mainly arises from object exploration tasks. Interestingly, the importance of the LEC in high memory load conditions has been described in a recognition task involving odors (Ku et al., 2017) and in rat trace eyeblink conditioning (Morrissey et al., 2012). Whether task demand or task complexity is a relevant notion to account for a potential different involvement of the two sub-regions in navigation and between navigation and more spontaneous exploration tasks is an open question.

One possibility to account for spared abilities in “simple” environments is that the functions are supported by other brain circuits that do not involve the EC, and that are not identified. Alternatively, it is possible that spatial and non-spatial processing is maintained by some compensation processes between the two areas. For example, perirhinal and postrhinal inputs targeting both the LEC and the MEC, may convey spatial and non-spatial information to the non-lesioned area. Such inputs may be sufficient to support spatial and non-spatial processing in a simple environment. In conditions of “complex” environment, spatial processing is undertaken primarily by the MEC and the LEC processes associations between object identity and contextual information. This suggests that the MEC and the LEC tightly cooperate and work in a flexible manner to integrate the “what” and “where” information in episodic memory upstream the hippocampus. The neural substrates underlying such flexibility within the EC circuits are largely unknown. In that regard, it is interesting to note that entorhinal grid cell activity is more flexible than initially postulated, and may combine both idiothetic and allothetic information (Derdikman et al., 2009; Barry et al., 2012). Such a combination is poorly understood however but this is undoubtedly a crucial issue that would provide the keys to a unitary model of EC functioning. Further evidence of flexibility within the EC circuits would be supported by the existence of intrinsic MEC-LEC neuroanatomical and functional interactions. The complex organization of the EC also needs to be studied in the context of the whole EC-hippocampal system as the function of the EC is tightly dependent of that of the hippocampus and vice versa (Brun et al., 2008; Bonnevie et al., 2013). Deciphering the processing of spatial information and episodic memory in the brain will be achieved only if we take into account the whole entorhinal hippocampal circuit.

## Author Contributions

ES and FS have both participated in the writing of the article.

## Conflict of Interest Statement

The authors declare that the research was conducted in the absence of any commercial or financial relationships that could be construed as a potential conflict of interest.
